# A Feature Fusion Predictor for RNA Pseudouridine Sites with Particle Swarm Optimizer Based Feature Selection and Ensemble Learning Approach

**DOI:** 10.3390/cimb43030129

**Published:** 2021-11-01

**Authors:** Xiao Wang, Xi Lin, Rong Wang, Nijia Han, Kaiqi Fan, Lijun Han, Zhaoyuan Ding

**Affiliations:** 1 School of Computer and Communication Engineering, Zhengzhou University of Light Industry, Zhengzhou 450002, China; xlin@xwanglab.com (X.L.); rwang@xwanglab.com (R.W.); njhan@xwanglab.com (N.H.); ljhan@xwanglab.com (L.H.); zyding@xwanglab.com (Z.D.); 2 School of Material and Chemical Engineering, Zhengzhou University of Light Industry, Zhengzhou 450002, China; benlto@163.com

**Keywords:** RNA pseudouridine sites, feature fusion, particle swarm optimization, feature selection, ensemble learning

## Abstract

RNA pseudouridine modification is particularly important in a variety of cellular biological and physiological processes. It plays a significant role in understanding RNA functions, RNA structure stabilization, translation processes, etc. To understand its functional mechanisms, it is necessary to accurately identify pseudouridine sites in RNA sequences. Although some computational methods have been proposed for the identification of pseudouridine sites, it is still a challenge to improve the identification accuracy and generalization ability. To address this challenge, a novel feature fusion predictor, named PsoEL-PseU, is proposed for the prediction of pseudouridine sites. Firstly, this study systematically and comprehensively explored different types of feature descriptors and determined six feature descriptors with various properties. To improve the feature representation ability, a binary particle swarm optimizer was used to capture the optimal feature subset for six feature descriptors. Secondly, six individual predictors were trained by using the six optimal feature subsets. Finally, to fuse the effects of all six features, six individual predictors were fused into an ensemble predictor by a parallel fusion strategy. Ten-fold cross-validation on three benchmark datasets indicated that the PsoEL-PseU predictor significantly outperformed the current state-of-the-art predictors. Additionally, the new predictor achieved better accuracy in the independent dataset evaluation—accuracy which is significantly higher than that of its existing counterparts—and the user-friendly webserver developed by the PsoEL-PseU predictor has been made freely accessible.

## 1. Introduction

With the next generation of sequencing technology rapidly developing, the identification of RNA pseudouridine sites has gradually become one of the most significant areas in transcriptome research. Pseudouridine sites have been found in various RNAs, including tRNA, mRNA, snRNA, snoRNA, and rRNA. [1]. Pseudouridine sites are considered to be among the most basic RNA modification sites found in prokaryotes and eukaryotes [2]. As one of the most enriched post-transcriptional modifications, pseudouridylation plays an important role in the structure, function, and metabolism of RNA [3,4,5,6]. Therefore, the study of pseudouridine modification sites is very important in further revealing their related biological principles: for instance, their role in stress response and in stabilizing RNA [7,8]. However, genome-wide analysis experiments are labor intensive, time consuming, and costly [9,10,11,12]. Considering the rapidly increasing amount of data generated in the post-genome era, it is necessary to build computational tools that can identify pseudouridine sites efficiently. In recent years, several fast and inexpensive methods for predicting RNA pseudouridine site have emerged [13,14,15,16,17,18].

Li et al. used the PUS specificity in *H. sapiens* and in *S. cerevisiae* to build the first computational tool, PPUS, to identify pseudouridine sites [13]. Later, inspired by these works, Chen et al. developed an iRNA-PseU predictor using an SVM model [14]. In addition, He et al. proposed the PseUI [15] predictor, for further enhancing the accuracy of the identification of RNA pseudouridine sites. Following this, Tahir et al. used convolutional neural networks to design a new predictor, iPseU-CNN [16]. Liu et al. used the eXtreme Gradient Boosting method for RNA pseudouridine site prediction, the predictor of which is called XG-PseU [17]. Lv et al. built a new predictor, RF-PseU [18], based on the random forest algorithm, which has achieved state-of-the-art results. Mu et al. proposed an effective layered ensemble model, designated as the iPseU-Layer, for the identification of RNA pseudouridine sites [19]. Song et al. used traditional sequence features and 42 additional genomic features to build a high-accuracy predictor in PIANO [20]. Recently, Aziz et al. proposed a multi-channel convolutional neural network using binary encoding to construct a predictor for identifying RNA pseudouridine sites [21]. These existing predictors have achieved fine results, but they were not constructed in a systematic way for the exploration and extraction of different types of RNA sequence descriptors. Moreover, concerning redundant and invalid features, greedy algorithms (e.g., incremental feature selection methods (IFS) or sequential forward selection strategies (SFS)) are usually employed to filter the predictors’ features. Therefore, there is still significant room for improvement in the accuracy of identification and the generalization ability of predictors. Additionally, these predictors cannot identify RNA sequences of indeterminate length, which need to be manually cut before batch identification can be performed.

**Figure 1:**
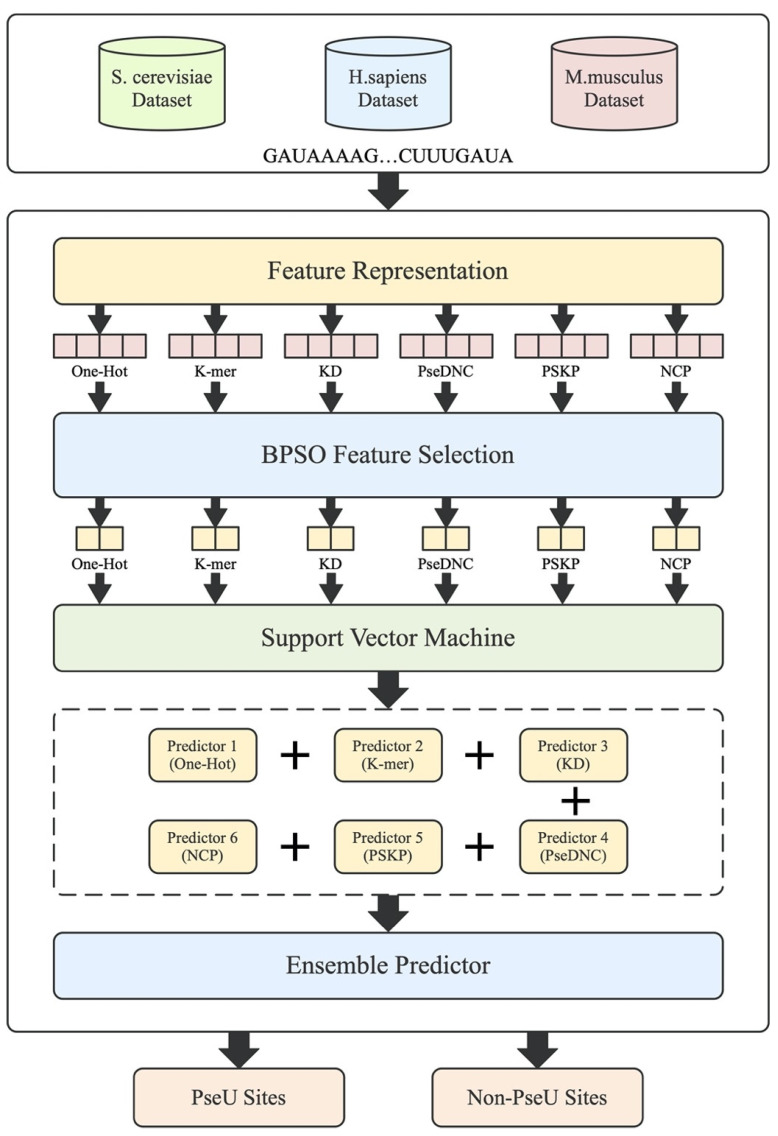
The framework of the proposed PsoEL-PseU predictor. The RNA sequences in the dataset are represented by feature descriptors, followed by filtering using the BPSO algorithm; finally, the optimal subset of feature descriptors is used to train the predictors and assemble the six basic predictors to construct the PsoEL-PseU predictor.

Motivated by this, a novel feature fusion predictor called PsoEL-PseU is proposed for pseudouridine site identification in this paper. Firstly, we systematically determined and analyzed six different types of feature descriptors. To make full use of these feature descriptors, we used a thorough search selection algorithm (binary particle swarm optimization algorithm) to eliminate a large number of redundant and invalid features, as compared with the greedy sequence algorithms used in the aforementioned papers. Afterwards, a parallel fusion strategy was used to fuse these six optimal feature subsets, which further improved the accuracy in identifying pseudouridine sites. Finally, in our predictor webserver, we used a sliding window approach to solve the problem of identifying the pseudouridine site locations of indeterminate length in RNA sequences. The modeling framework is shown in Figure 1. For the improvement of the feature representation ability, a binary particle swarm optimizer was used to capture the optimal feature subset for each of the six feature descriptors (one-hot encoding, k-mer nucleotide frequency, k-nucleotide density, pseudo dinucleotide composition [22], position-specific k-nucleotide propensity [23], and nucleotide chemical property). For the parallel fusion strategy, we used a majority voting strategy for the fusion of the six feature descriptors. Ten-fold cross-validation on three benchmark datasets indicated that the PsoEL-PseU predictor significantly outperformed other current state-of-the-art predictors. Additionally, the new predictor achieved better accuracy in independent dataset evaluations, with significantly higher accuracy than its existing counterparts. PsoEL-PseU is expected to become a useful tool for identifying RNA pseudouridine sites. The user-friendly webserver that has been developed for the PsoEL-PseU predictor can be accessed for free via the following link: http://www.xwanglab.com/PsoEL-PseU/Server (accessed on 19 July 2021).

## 2. Materials and Methods

### 2.1. Benchmark Datasets

This study used three different species benchmark datasets and two different species independent test datasets which were used in iRNA-PseU [14] to perform a comprehensive and unbiased comparison. The three benchmark datasets collected from the *S. cerevisiae*, *H. sapiens*, and *M. musculus* species consist of 628, 990, and 944 RNA sequences, respectively. They have the same number of pseudouridine site sequences and non-pseudouridine site sequences. The RNA sequences in the *S. cerevisiae* dataset contain 31 nucleotides, while the RNA sequences in the *H. sapiens* and *M. musculus* datasets both contain 21 nucleotides. In addition, two independent test datasets, only from the *S. cerevisiae* and *H. sapiens* species, both contain 100 pseudouridine site sequences and 100 non-pseudouridine site sequences. The benchmark datasets and independent test datasets can be downloaded from the webserver via http://www.xwanglab.com/PsoEL-PseU/Download (accessed on 19 July 2021).

### 2.2. Feature Representation

#### 2.2.1. One-Hot Encoding

One-hot encoding encodes the original RNA sequence directly, thus retaining the most primitive and simple sequence information. It mainly uses the N-bit state register to encode N states. Each state has an independent register bit, and only one bit is valid at any time. Therefore, the 4 nucleotides and 16 dinucleotides in RNA can be encoded by 0 and 1, and thus A, U, C, G, AA, AU, …GC, and GG can be converted to (1, 0, 0, 0), (0, 1, 0, 0), (0, 0, 1, 0), (0, 0, 0, 1), (0, 0,…0,1), (0, 0,…1, 0), …(0, 1,…0, 0), and (1, 0,…,0, 0), respectively. Therefore, if the length of the RNA sequence is λ, the RNA sequence can be represented by a (20λ−16)-dimensional (4λ-dimensional + 16(λ−1)-dimensional) vector.

#### 2.2.2. K-Mer Nucleotide Frequency (K-Mer)

K-mer nucleotide frequency (K-mer) is the most common feature descriptor, in which the RNA sequences are represented as the occurrence frequency of k neighbor nucleic acids. In this study, we chose k that is equal to 1 and 2, which means that the method will extract 20-dimensional features (4-dimensional + 16-dimensional). Because it contains the frequencies of the mononucleotides and dinucleotides for each RNA sequence, they are A, U, C, G, AA, AU…GC.

#### 2.2.3. K-Nucleotide Density (KD)

For further obtaining the frequency information of RNA sequences, this study adopted the k-density method (KD) to measure the correlation between positions and frequencies of k neighboring residues in the RNA sequences. The density of $d_{i}$ can be represented by the following Equation (1):

(1) $$d_{i}=\frac{1}{\left|N_{i}\right|}\overset{L}{\underset{j=1}{\sum }}f\left(n_{j}\right)\;,\;\;\;\;\;\;f\left(n_{j}\right)=\{\begin{array}{c} \;1\;\;\;\;\;\;if\;\;\;n_{j}=q\\ \;0\;\;\;\;\;\;if\;\;\;n_{j}\neq q \end{array}$$

where $\left|N_{i}\right|$ denotes the length from the first nucleotide to the current nucleotide position; *L* denotes the sequence length; and q is a symbol of {A, U, C, G}. In similar situations, we chose k that is equal to 1 and 2.

#### 2.2.4. Pseudo Dinucleotide Composition (PseDNC)

A vector defined in a discrete space may completely miss all the sequence order or pattern information. Therefore, to obtain more long-range sequence information, this study adopted the pseudo dinucleotide composition (PseDNC) descriptor to make up for the missing information. This type of pseudo composition can still continuously feed the global sequence order information and the local sequence order information into the feature descriptor for RNA sequences. Three physicochemical properties, namely, free energy, stacking energy, and hydrophilicity, are used to generate feature descriptors composed of pseudo dinucleotides. The details of the description can be found in [22].

#### 2.2.5. Position-Specific K-Nucleotide Propensity (PSKP)

After extracting information on the frequency (k-mer), density (KD), order (PseDNC), and original sequence representation (One-Hot) of a single RNA sequence, we hoped to obtain the global nucleotide information of the homotypic sequences. The position-specific nucleotide propensity [23] descriptor represents a more effective way to obtain global information, by calculating the differences in the frequency of nucleotides in specific locations between pseudouridine site and non-pseudouridine site sequences in RNA sequences. Similarly, we extracted frequency information from homotypic sequences at specific locations of mononucleotides and dinucleotides.

#### 2.2.6. Nucleotide Chemical Property (NCP)

To extract some intrinsic information between nucleotides, in this study, we adopted the nucleotide chemical property (NCP) method. This method divides the four types of nucleotides into three categories based on the functional groups and hydrogen bonds of the ring structures. Therefore, a 3-dimensional vector representing a given RNA sequence is used to quantify these chemical properties, ($x_{i}$, $y_{i}$, $z_{i}$), where $x_{i}$, $y_{i}$, and $z_{i}$ are represented as follows:

(2) $$x_{i}=\{\begin{array}{c} 1,\;if\;N_{i}\in \left\{A,G\right\}\\ 0,\;if\;N_{i}\in \left\{C,U\right\} \end{array}\;y_{i}=\{\begin{array}{c} 1,\;if\;N_{i}\in \left\{A,C\right\}\\ 0,\;if\;N_{i}\in \left\{G,U\right\} \end{array}\;z_{i}=\{\begin{array}{c} 1,\;if\;N_{i}\in \left\{A,U\right\}\\ 0,\;if\;N_{i}\in \left\{C,G\right\} \end{array}$$

Among them, $x_{i}$ encodes nucleotides by ring structure; $y_{i}$ encodes nucleotides by functional groups; and $z_{i}$ encodes nucleotides by the strength of hydrogen bonds. For a detailed description, refer to [14]. Thus, according to Equation (2), nucleotide *A* can be represented as (1, 1, 1), *U* as (0, 0, 1), *C* as (0, 1, 0), and *G* as (1, 0, 0).

### 2.3. Feature Selection

To improve the feature representation ability of the genome sequences, selecting smaller feature subsets to reduce redundancy may improve the generalization ability of the predictor. Therefore, it is essential to perform feature selection on original features. In the field of bioinformatics, the most common feature selection method is the sequential algorithm (e.g., incremental feature selection methods (IFS) [24,25,26,27] or sequential forward selection strategies (SFS) [28,29,30]). The essence of the sequential algorithm is to use the idea of the greedy strategy, which tends to fall into the local optimal. Therefore, this study introduces a heuristic search algorithm that enables a thorough search for genome sequence features in the feature space.

#### Binary Particle Swarm Optimization (BPSO)

An improved binary particle swarm optimization (BPSO) algorithm [31] is introduced for feature selection of genomic sequence information. BPSO is a discrete population-based optimization computational tool. In this case, each population has multiple particles, and each particle has its own position x and velocity v. The position x represents the potential solution to the problem. The velocity v determines the direction of movement of the particles. The velocity v is determined by the previous historical optimal position of each particle and the global optimal position of the whole population thus far. Therefore, the particle moves in the direction of its previous optimal position and the global optimal position in each iteration, finding the best solution to the problem. For a detailed description of the formulation, refer to Supplementary Data Equations (S1)–(S4).

**Figure 2:**
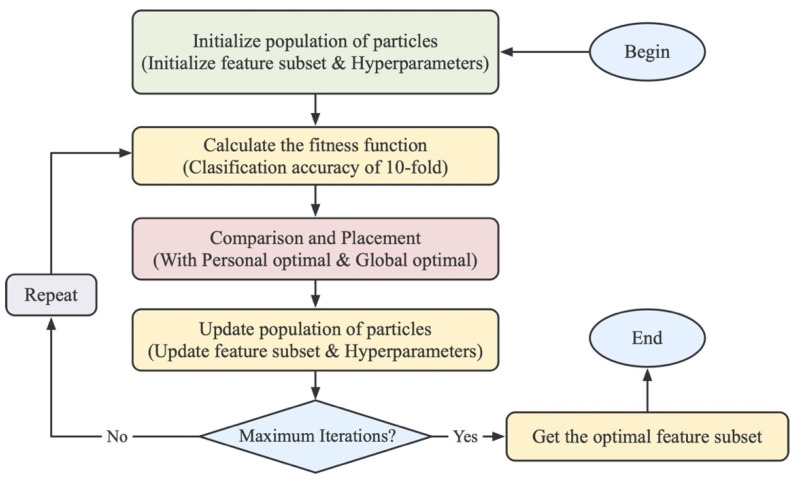
Flow chart of the BPSO algorithm. The particles are first initialized, and then the fitness value of each particle is calculated. The fitness value is used to iteratively update the velocity and position, in order to find the optimal feature subset of feature descriptors.

In this study, each particle consisted of two parts, where the first part represents the result of feature selection. Its length is equal to the number of original features. The value of 0 or 1 of the particle position is used to indicate whether the feature at the corresponding position is selected. The second part represents the result of the hyperparameter combination trained by the support vector machine, consisting of 10 binary bits, which can represent a total of 1024 hyperparameter combinations. Finally, in order to obtain more efficient features, the classification accuracy of the ten-fold cross-validation of the identified pseudouridine sites was used as the fitness value to ensure that the population particles move towards a high classification accuracy. The detailed flow chart is shown in Figure 2.

### 2.4. Support Vector Machine (SVM)

Support vector machines (SVMs) are dichotomous models. They are widely used for classification problems in the field of bioinformatics, with very effective results. Their basic model is a linear classifier defined with the largest spacing between two categories (positive and negative) in the feature space. Furthermore, SVMs also include kernel techniques, which essentially make them a nonlinear classifier. This study used the radial basis function (RBF) as the kernel function of the SVM and implemented the SVM algorithm in the scikit-learn (v 0.20.0) library in Python3. There are some hyperparameters for the RBF kernel function, namely, the kernel parameter γ and the regularization parameter C. To obtain a more accurate model, we set these hyperparameters with a larger search space, and the search ranges for both parameters are shown belowin Equation (3), where there are geometric sequences with a common ratio of 2:

(3) $$\left\{ \begin{array}{cc} 2^{-16}⩽C⩽2^{15} & \mathrm{common}\;\mathrm{ration}\;\mathrm{of}\;2\\ 2^{-16}⩽\gamma ⩽2^{15} & \mathrm{common}\;\mathrm{ration}\;\mathrm{of}\;2 \end{array}\right.$$

### 2.5. Fusion Strategy

To enhance the predictor performance and obtain stronger robustness, two fusion strategies can be considered: one is serial fusion, and the other is parallel fusion. The serial fusion strategy means directly serially merging the six optimal genomic sequence features determined by the BPSO algorithm, and using the SVM to relearn and find the optimal decision boundaries for identifying pseudouridine sites. The parallel fusion strategy concerns the fusion of the basic predictors of the six optimal genomic sequence features determined by the BPSO algorithm, and the fusion approach generally adopts an ensemble approach.

In this study, a parallel fusion strategy was used to perform information fusion. The reason for this is that the results of pre-experiments indicated that the parallel fusion strategy will be better than the serial fusion strategy. We speculated that one of the biggest reasons why parallel fusion is better than serial fusion is that while using BPSO to optimize the features, we also select the hyperparameters of the classifier, meaning that the optimized features often have widely different classification boundaries. If multiple genomic sequence features with different classification boundaries are directly fused in series, it will be relatively difficult for the new classifier to relearn and describe the classification boundaries of these different genomic sequence features simultaneously. In contrast, the parallel fusion strategy is a direct fusion of the basic predictors of the selected optimal genomic sequence features, and the different classification boundaries often have complementary effects, thus making parallel fusion more straightforward and effective compared to serial fusion. Therefore, this study finally adopted a parallel fusion strategy for information fusion, and the parallel fusion method used an ensemble learning approach of the majority voting strategy to fuse the six basic predictors.

### 2.6. Model Evaluation 

In the field of bioinformatics and recent studies, four metrics are used to evaluate the quality of predictors. They are specificity (SP), sensitivity (SN), accuracy (ACC), and Mathew’s correlation coefficient (MCC). The formulas are as follows (Equations (4)–(7)):

(4) $$\mathrm{SN}=\frac{\mathrm{TP}}{\mathrm{TP}+\mathrm{FN}}$$

(5) $$\mathrm{SP}=\frac{\mathrm{TN}}{\mathrm{TN}+\mathrm{FP}}$$

(6) $$\mathrm{ACC}=\frac{\mathrm{TP}+\mathrm{TN}}{\mathrm{TP}+\mathrm{TN}+\mathrm{FN}+\mathrm{FP}}$$

(7) $$\mathrm{MCC}=\frac{\mathrm{TP}\times \mathrm{TN}-\mathrm{FP}\times \mathrm{FN}}{\sqrt{\left(\mathrm{TP}+\mathrm{FN}\right)\left(\mathrm{TP}+\mathrm{FP}\right)\left(\mathrm{TN}+\mathrm{FP}\right)\left(\mathrm{TN}+\mathrm{FN}\right)}}$$

where TP (true positive) is the observation of pseudouridine sites where they were predicted to be pseudouridine sites; TN (true negative) is the observation of non-pseudouridine sites where they were predicted to be non-pseudouridine sites; FN (false negative) is the observation of pseudouridine sites, but they were predicted to be non-pseudouridine sites; and FP (false positive) is the observation of non-pseudouridine sites, but they were predicted to be pseudouridine sites.

Hence, SN is the probability of obtaining a correct prediction of pseudouridine sites. SP is the probability of obtaining a correct prediction of non-pseudouridine sites. ACC represents the accuracy of the overall RNA sequence site prediction. Because the MCC takes into account true positive, false positive, true negative, and false negative observations, it is often seen as a measure of balance.

## 3. Results and Discussion

### 3.1. Determining the Optimal Feature Subset

In this study, the binary particle swarm optimization (BPSO) algorithm was used to select the optimal feature subset. The position and velocity dimensions of the BPSO algorithm were equal to the feature and hyperparameter dimensions. Based on preliminary experiments, we found that when the number of particles in the population was between 60 and 80, the global position was updated using Supplementary Data Equation (S3). The accuracy value almost ceased to improve when the number of iterations reached 150 to 200, and the particles entered a blind search state. Therefore, in order to accelerate convergence, we replaced Supplementary Data Equation (S3) with Supplementary Data Equation (S4) to update the global positions. Then, within approximately 100 iterations, the velocity of all particles almost reached zero, and they were no longer moving. Thus, we combined the computational and time costs for consideration, the particle swarm selected 80 particles to ensure sufficient diversity in the population, and we set 300 iterations to ensure a sufficient global search. The first 200 iterations used Supplementary Data Equation (S3) to update the global position, and the last 100 iterations used Supplementary Data Equation (S4) to update the global position; the velocity range set in this study was (−6, 6).

**Figure 3:**
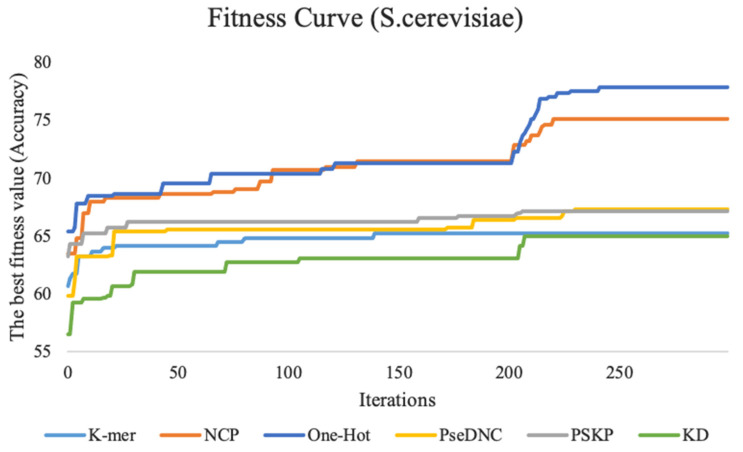
The fitness curves of six feature descriptors on the *S. cerevisiae* dataset. K-mer represents the fitness curve for nucleotide frequency, KD represents the fitness curve for nucleotide density, PseDNC represents the fitness curve for pseudo dinucleotide composition, PSKP represents the fitness curve for position-specific k-nucleotide propensity, and NCP represents the fitness curve for nucleotide chemical property. Additionally, the vertical coordinates represent the accuracy, and the horizontal coordinates represent the number of iterations.

This study performed feature tuning experiments on three benchmark datasets. To show the experimental process more intuitively, we plotted the fitness curve to visualize the global optimal accuracy in the iterative process. Figure 3 shows the fitness curves of the six feature descriptors on the *S. cerevisiae* dataset.

The fitness curve of the k-mer feature descriptor stabilizes as the number of iterations reaches 150. The reason for this result may be that the feature dimension of this method is smaller, with only 20 dimensions, the particle swarm used in this study is relatively large, and the search is relatively sufficient. Therefore, it is easy to achieve convergence and find the global optimal feature subset. The ten-fold cross-validation accuracy of the features for the PseDNC, KD, and PSKP feature descriptors is improved by a total of 7.5%, 8.4%, and 4% with an increasing number of iterations compared with the first iteration, respectively. The other two feature descriptors (NCP and One-Hot) contain a large number of discrete and redundant features after 0/1 coding. After full screening and filtering by the BPSO algorithm, the accuracy of the ten-fold cross-validation is fully improved, which increases by 12.4% and 11.6% compared with the first iteration. For the H. sapiens dataset and M. musculus dataset, their performance trends are similar to the *S. cerevisiae* dataset. For details, please refer to Supplementary Data Figure S1.

In summary, as the number of iterations increases, an increase in the accuracy of the ten-fold cross-validation of the fitness curves and the basic predictor on the three species can be observed. This basically indicates that the larger the feature dimension, the more obvious the improvement effect, which also shows that the BPSO algorithm plays a very effective role.

### 3.2. Comparative Analysis on Different Feature Selection Methods

At the same time, to further demonstrate the superiority of the BPSO feature selection algorithm, this study compared the BPSO feature selection method with the incremental feature selection (IFS) method on the three benchmark datasets.

**Figure 4:**
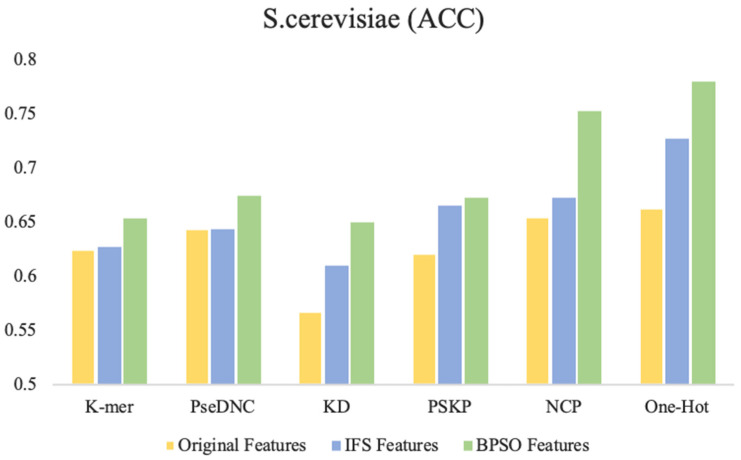
Comparison of performance between different feature selection methods and BPSO method. The Original Features represent all the features without feature selection, the IFS Features represent the optimal subset of features selected using incremental feature selection methods, and the BPSO Features represent the optimal subset of features selected using the binary particle swarm optimization algorithm.

Figure 4 shows the accuracy of the original features, IFS features (selected by the IFS method), and the BPSO features (selected by the BPSO method) trained on the SVM. It can be intuitively found that in the six feature descriptors of the *S. cerevisiae* dataset, the accuracy of the IFS method is improved to various degrees compared with the original feature. However, compared with the IFS method, the BPSO algorithm is further improved. In particular, the accuracy of the BPSO feature selection method compared with the IFS feature selection method is increased by 8% and 5.2% with NCP and One-Hot, respectively. Moreover, compared with the original features, the accuracy is increased by 9.8% and 11.7%. 

In terms of feature size, the BPSO method selects a relatively balanced feature size, while the features selects by IFS have two extremes. One extreme is similar to PseDNC on the *S. cerevisiae* dataset, where the feature finally selected the full set as the optimal feature subset. The other is similar to k-mer on the *H. sapiens* dataset and KD on the *M. musculus* dataset. When the size equals 4 and 3, the accuracy of the predictors is the highest, and the predictors finally select relatively low-dimensional features as the optimal feature subset. In the later experiments, we found that the overfitting probability of these low-dimensional features was greater than the probability of higher-dimensional features. The detailed comparison results are shown in Supplementary Data Table S1. 

Through observation, it was found that compared to other feature selection strategies on the three benchmark datasets, the key features captured using the BPSO feature selection strategy performed best with the One-Hot and NCP feature descriptors. We attributed this to two factors: (1) The One-Hot feature descriptor retains the most primitive and basic RNA sequence information, while the NCP feature descriptor contains sufficient structural information. These feature descriptors have strong representation capability by themselves. (2) The BPSO method searches for the optimal feature subset in the global space, and the search is relatively more thorough compared to the greedy search strategy in the IFS method. Compared with the other feature descriptors, the One-Hot and NCP feature descriptors have larger dimensionality after encoding, and they have a larger feature space and also contain more discrete and redundant features. Therefore, the performance of these two feature descriptors is the best. This also indicates the ability of the BPSO feature selection algorithm to capture high-dimensional key features compared to the IFS feature selection method.

### 3.3. Ensemble Predictor

Considering that an ensemble of multiple predictors generally provides a better performance, in this study, we used a parallel fusion strategy for information fusion, and the fusion approach used the ensemble approach of the majority voting strategy. The experimental results indicate that the ensemble predictor is further improved on the three benchmark datasets, as shown in Table 1. Compared with the six basic predictors, the accuracy of the *H. sapiens*, *S. cerevisiae*, and *M. musculus* datasets are greatly improved, increasing by 2.3%~8.6%, 2.4%~15.3%, and 1.5%~16.3%, respectively.

**Figure 5:**
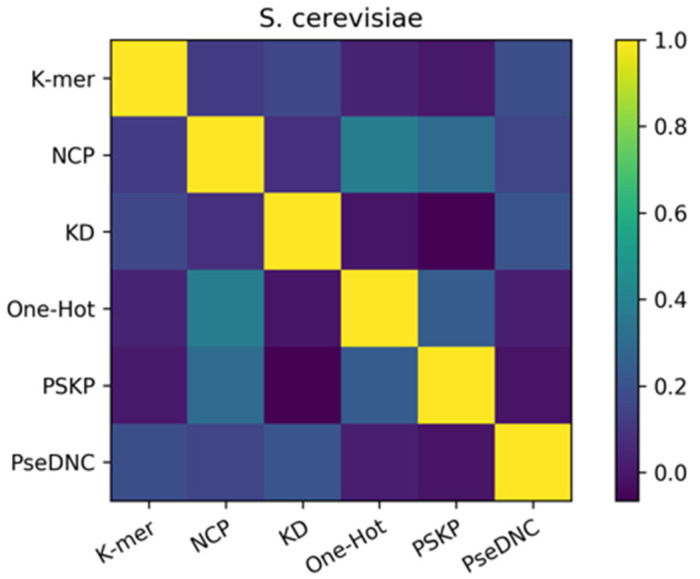
Pearson correlation coefficients of the feature descriptors on the *S. cerevisiae* dataset. The shades of color indicate the strength of similarity between the six basic predictors, with the darker color indicating weaker correlations.

To elucidate the reasons for the success of the ensemble approach, we performed a diversity analysis of the six basic predictors using the Pearson correlation coefficient approach. In this study, the Pearson correlation coefficients among the six features were calculated separately using the results of the six basic predictors and are shown as a heatmap. Figure 5 represents the heatmap of the *S. cerevisiae* dataset, where the shades of color indicate the strength of similarity between the six basic predictors, with the darker color indicating weaker correlations. At the same time, from Figure 5, we know that the final result of the ensemble method is effective because of the ensemble of heterogeneous predictors. Therefore, the Pearson correlation coefficient was calculated on the predictions of the six basic predictors to reflect the differences between the six basic predictors, as a way of exploring why the ensemble is effective. It can be seen from the figure that the Pearson correlation coefficients between the six features are all lower than 0.5. Among them, the correlation values of the PSKP and k-mer features, One-Hot and KD features, and PseDNC and PSKP features with the darkest colors are all lower than 0.2. This indicates that there is a very weak correlation, or strong heterogeneity, between the nucleotide sequence information extracted from the global RNA sequences with the nucleotide frequency and nucleotide order extracted from individual RNA sequences, and there is also a weak correlation between the original RNA sequence information and the nucleotide density. In addition, it can be found from the depth of color that there are at least three or more features with great differences in the six feature descriptions. Therefore, this basically reflects the complementary effect between these different types of feature descriptors, which is also the reason for the improvement after forming the ensemble. The provided Supplementary Data Figure S2 also illustrates the strong differences between the six features of the *H. sapiens* dataset and the *M. musculus* dataset.

### 3.4. Comparison with State-of-the-Art Predictors

**Figure 6:**
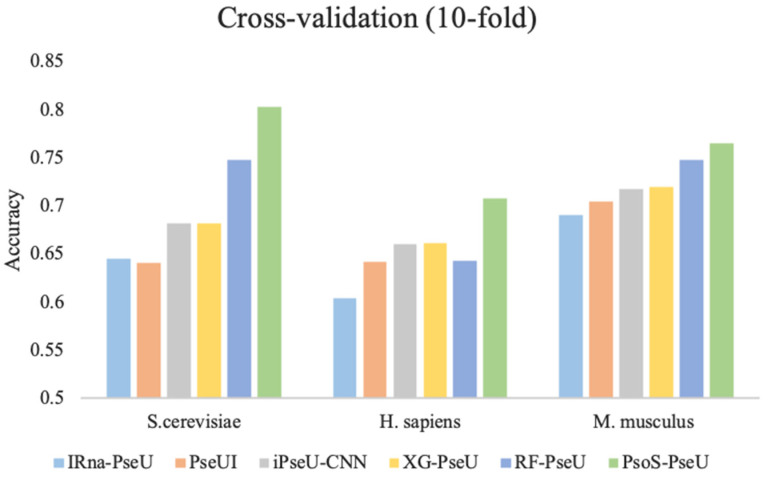
Performance evaluation of the PsoEL-PseU predictor and five state-of-the-art predictors on three benchmark datasets. The legend represents the names of the six state-of-the-art predictors.

To further demonstrate the prediction performance of our proposed predictor, PsoEL-PseU, in this study, we compared PsoEL-PseU with the existing state-of-the-art predictors including iRNA-PseU [14], PseUI [15], iPseU-CNN [16], XG-PseU [17], and RF-PseU [18] on the three benchmark datasets using ten-fold cross-validation. Figure 6 shows the comparative results for the state-of-the-art predictors with PsoEL-PseU. In the *H. sapiens* and *S. cerevisiae* datasets, the accuracy of the PsoEL-PseU predictor is 70.8% and 80.3%, which is improved by 6.5% and 5.5% compared with the best predictor (RF-PseU), respectively. As for the *M. musculus* dataset, the accuracy of the PsoEL-PseU predictor compared with the existing state-of-the-art predictors also exceeds the range of 1.7–7.4%.

In general, the result of the PsoEL-PseU predictor using ten-fold cross-validation on the three benchmark datasets is better than that of the state-of-the-art predictors in the four metrics, including ACC, MCC, SN, and SP. More detailed results of the evaluation metrics are provided in Supplementary Data Table S2. Additionally, ultimately, it can be found that the overall performance of PsoEL-PseU is markedly better than that of other state-of-the-art predictors. This indicates that PsoEL-PseU can identify pseudouridine sites with significantly better accuracy than the existing state-of-the-art predictors.

### 3.5. Comparative Analysis on Independent Datasets

To demonstrate the generalization ability of our predictor for identifying pseudouridine sites, this study validated them on independent datasets built by Chen et al. [14]. These include two species datasets, *H. sapiens* and *S. cerevisiae*. Through applying the predictor PsoEL-PseU to the two independent test datasets, four metrics were computed. Finally, we compared the results with those of seven state-of-the-art predictors, including iR-NA-PseU [14], PseUI [15], iPseU-CNN [16], XG-PseU [17], RF-PseU [18], iPseU-Layer [19], and iPseUMultiCNN [21], and the detailed comparison can be found in Table 2. It can be observed that the PsoEL-PseU predictor remarkably outperforms the state-of-the-art predictors on the two independent datasets. In particular, for the *S. cerevisiae* dataset, the ACC value of the PsoEL-PseU predictor exceeds that of the current best predictor (RF-PseU) by 5%, and the MCC value is increased by more than 10%. Additionally, compared to the newer layered ensemble predictor iPseU-Layer and the multi-channel convolutional neural network predictor iPseUMultiCNN, the ACC values also increase by 9.5% and 6%. However, on the *H. sapiens* dataset, our predictor is only slightly improved compared to the RF-PseU and iPseUMultiCNN predictors, where the ACC value only increases by 0.5% and 1.5%. Additionally, on the SN metric, our predictor is outperformed by the iPseU-CNN and RF-PseU predictors by 1.7% and 2%. On the SP metric, our predictor is again outperformed by 4% by the iPseU-Layer predictor. However, on the MCC metric, which measures the overall predictive balance, our predictor is optimal, with MCC values improving by 1% to 8% compared to RF-PseU, iPseU-Layer, and iPseUMultiCNN. For comprehensive comparison, the average metrics for the two independent datasets were computed. For the PsoEL-PseU predictor, the average values for ACC, MCC, SN, and SP reach 0.788, 0.575, 0.795, and 0.78, respectively. These results indicate that PsoEL-PseU exceeds the existing predictors in all evaluation metrics. 

According to the ten-fold cross-validation and independent test evaluation, PsoEL-PseU outperforms the existing state-of-the-art predictors. We attributed the success of PsoEL-PseU to three factors: (1) Previous feature descriptors are relatively simple, whereas this study systematically and comprehensively explored different types of feature descriptors and determined six feature descriptors with various properties. (2) This study used the BPSO algorithm to determine the optimal genome sequence feature for each basic predictor. Since the BPSO algorithm searches for the optimal feature subset in the global space, the search is relatively more thorough compared to the greedy search strategy. (3) The ensemble predictor was developed by combining the six different RNA basic predictors, which further enhanced the robustness of the predictor. As a result, PsoEL-PseU is anticipated to be an essential computational tool for the identification of pseudouridine sites in *H. sapiens*, *S. cerevisiae*, and *M. musculus*. Although these factors led to a decent improvement in our proposed predictor, especially on the *S. cerevisiae* species dataset, in the case of the *H. sapiens* species dataset, the improvement was slight. We speculated that this may be a result of the different RNA sequence lengths in the benchmark datasets, with each RNA sequence possessing a length of 41 bp for the *S. cerevisiae* dataset and only 21 bp for the *H. sapiens* dataset. For longer sequences, five of the six feature representations covered in this study are able to extract more features (PseDNC, KD, PSKP, NCP, One-Hot). Thus, the *S. cerevisiae* species dataset has a larger feature space, and its performance will clearly be more significantly improved if a more adequate and thorough search is performed to filter out the optimal feature subset using heuristic search methods.

## 4. Conclusions

In this study, the PsoEL-PseU predictor was proposed as a novel feature fusion predictor for the prediction of pseudouridine sites. We constructed the PsoEL-PseU predictor by combining a binary particle swarm optimizer algorithm to capture the optimal subset of features with different types of feature descriptors, and then employed a parallel fusion strategy to fuse these features. In addition, we used a sliding window approach to solve the problem where the predictors cannot identify RNA sequences with indeterminate lengths. The constructed predictor showed a remarkable improvement in the average prediction accuracy compared to several current state-of-the-art predictors. However, due to the large number of updates and iterations required for feature selection, the computational cost of the particle swarm optimization algorithm is proportional to the dimensionality of the features. This inevitably results in more computational resources being consumed when constructing site identification problems for a new species. Moreover, all state-of-the-art predictors are currently modeled for single species or single sites, which results in unsatisfactory prediction effects for different species or sites. Inspired by this, we will attempt to use evolutionary algorithms which solve multi-objective optimization problems to solve different species or site prediction problems in the future. In addition, we will use more advanced deep learning techniques to excavate RNA sequence information and fuse more genomic features to further improve the predictor′s ability to identify RNA sites. Finally, a user-friendly webserver for the PsoEL-PseU predictor has been developed and can be accessed for free at http://www.xwanglab.com/PsoEL-PseU/Server (accessed on 19 July 2021).

## Figures and Tables

**Table 1:** The cross-validation scores of the ensemble predictor for three benchmark datasets.

Cross-Validation (Ten-Fold)				
Species	ACC	MCC	SN	SP
*H. sapiens*	0.708	0.42	0.669	0.747
*S. cerevisiae*	0.803	0.62	0.691	0.914
*M. musculus*	0.765	0.53	0.822	0.708

**Table 2:** Comparison of independent testing scores of PsoEL-PseU with existing state-of-the-art pseudouridine site predictors.

Species	Predictor	ACC	MCC	SN	SP
*H. sapiens*	IRna-PseU	0.65	0.3	0.60	0.70
	PseUI	0.655	0.31	0.63	0.68
	iPseU-CNN	0.69	0.40	0.777	0.608
	XG-PseU	0.675	/	/	/
	RF-PseU	0.75	0.5	0.78	0.72
	iPseU-Layer	0.71	0.43	0.63	0.79
	iPseUMultiCNN	0.74	0.48	0.73	0.75
	PsoEL-PseU	0.755	0.51	0.76	0.75
*S. cerevisiae*	IRna-PseU	0.60	0.20	0.63	0.57
	PseUI	0.685	0.37	0.65	0.72
	iPseU-CNN	0.735	0.47	0.686	0.778
	XG-PseU	0.71	/	/	/
	RF-PseU	0.770	0.54	0.75	0.79
	iPseU-Layer	0.725	0.45	0.68	0.77
	iPseUMultiCNN	0.76	0.53	0.80	0.73
	PsoEL-PseU	0.82	0.64	0.83	0.81

## Data Availability

Publicly available datasets were used in this study. The datasets can be obtained from the corresponding studies.

## Supplementary Materials

The following are available online at https://www.mdpi.com/article/10.3390/cimb43030129/s1.

## Abbreviations

BPSO	Binary particle swarm optimizer algorithm
SFS	Sequential forward selection strategies
IFS	Incremental feature selection strategies
ACC	Accuracy
MCC	Mathew’s correlation coefficient
SN	Sensitivity
SP	Specificity
K-mer	K-mer nucleotide frequency
PseDNC	Pseudo dinucleotide composition
KD	K-nucleotide density
PSKP	Position-specific k-nucleotide propensity
NCP	Nucleotide chemical property
