# Supplementary material for: A Feature Fusion Predictor for RNA Pseudouridine Sites with Particle Swarm Optimizer Based Feature Selection and Ensemble Learning Approach
Source: Curr Issues Mol Biol. 2021 Nov 1;43(3):1844–58. doi: 10.3390/cimb43030129 (PMC8929013; doi:10.3390/cimb43030129)
Supplement: Supplementary file 1 [file cimb-43-00129-s001.zip › cimb-1372780-supplementary.pdf]

A Feature Fusion Predictor for RNA Pseudouridine Sites with Particle Swarm Optimizer Based Feature Selection and Ensemble Learning Approach

Xiao Wang[1,*], Xi Lin[1] , Rong Wang[1], Nijia Han[1], Kaiqi Fan[2], Lijun Han[1] and Zhaoyuan Ding[1]

1 School of Computer and Communication Engineering, Zhengzhou University of Light Industry, Zhengzhou, 450002, China; xwang@xwanglab.com (X. W.); xlin@xwanglab.com (X. L.); rwang@xwanglab.com (R. W.); njhan@xwanglab.com (N. H); ljhan@xwanglab.com (L. H.); zyding@xwanglab.com (Z. D.)

2 School of Material and Chemical Engineering, Zhengzhou University of Light Industry, Zhengzhou 450002, China; benlto@163.com (K. F.)

* Correspondence: xwang@xwanglab.com (X. W.)

Supplementary Data

Binary particle swarm optimization (BPSO)

Particle swarm algorithm is a population-based optimization tool, which is inspired by social behavior among personal, and personal (particle) potential problem solutions move in an n-dimensional search space. Each particle records its previous optimal particle in *pbest* vector and records the global optimal particle so far of the entire population in *gbest* vector. All particles can share the search space information. Therefore, each particle moves in the direction of its previous optimal position (*pbest*) and the global optimal position (*gbest*) in each iteration. In each iteration, particles first calculate the velocity vector that determines the direction of its motion and then updates its position. The velocity update formula is Equation (S1):

$$v_{i,d}(t+1) = \omega \times v_{i,d}(t) + c_1 \times rnd(\,) \times \left(pbest_{i,d} - x_{i,d}(t)\right) + c_2 \times rnd() \times \left(gbest_d - x_{i,d}(t)\right) \quad \text{(S1)}$$

The positive constant ω is the weight of inertia, c_1, c_2 are personal and social learning factors, t is the iteration counter, i is the i-th particle, and d is the d-th dimension feature. $rnd()$ is a random number uniformly generated in the interval [0,1]. The new position of particles are calculated using the (4) (Equation (S2)):

$$x_{i,d}(t+1) = x_{i,d}(t) + v_{i,d}(t+1) \quad \text{(S2)}$$

To convert the continuous problem into the discrete problem. Kennedy and Eberhart introduced the sigmoid function to map the velocity in the interval [0,1] to express the probability of the binary bit is 1, the position update formula is changed to Equation (S3):

$$x_{i,d} = \begin{cases} 1, & if \quad rnd() < \dfrac{1}{1+e^{-v_{i,d}}} \\ 0, & if \quad rnd() \geqslant \dfrac{1}{1+e^{-v_{i,d}}} \end{cases} \quad \text{(S3)}$$

To avoid probability value of the velocity approaching 0 or 1, velocity is restricted to the $v_{i,d} \in [-v_{max}, v_{max}]$.

To increase the local search capability of the BPSO algorithm, after a certain number of iterations, this study quotes another strong discrete BPSO algorithm with local search capability to make it search the feature space more accurately. The location update formula is Equation (S4):

$$S(v_{i,d}) = \begin{cases} 1 - \dfrac{2}{1+e^{-v_{i,d}}}, & v_{i,d} < 0 \\ \dfrac{2}{1+e^{-v_{i,d}}} - 1, & v_{i,d} \geqslant 0 \end{cases}$$

$$x_{i,d} = \begin{cases} 1 & ,if\ \ v_{i,d} < 0\ \ and\ \ rnd() < S(v_{i,d}) \\ 0 & ,if\ \ v_{i,d} \geqslant 0\ \ and\ \ rnd() < S(v_{i,d}) \\ x_{i,d} & ,otherwise \end{cases} \tag{S4}$$

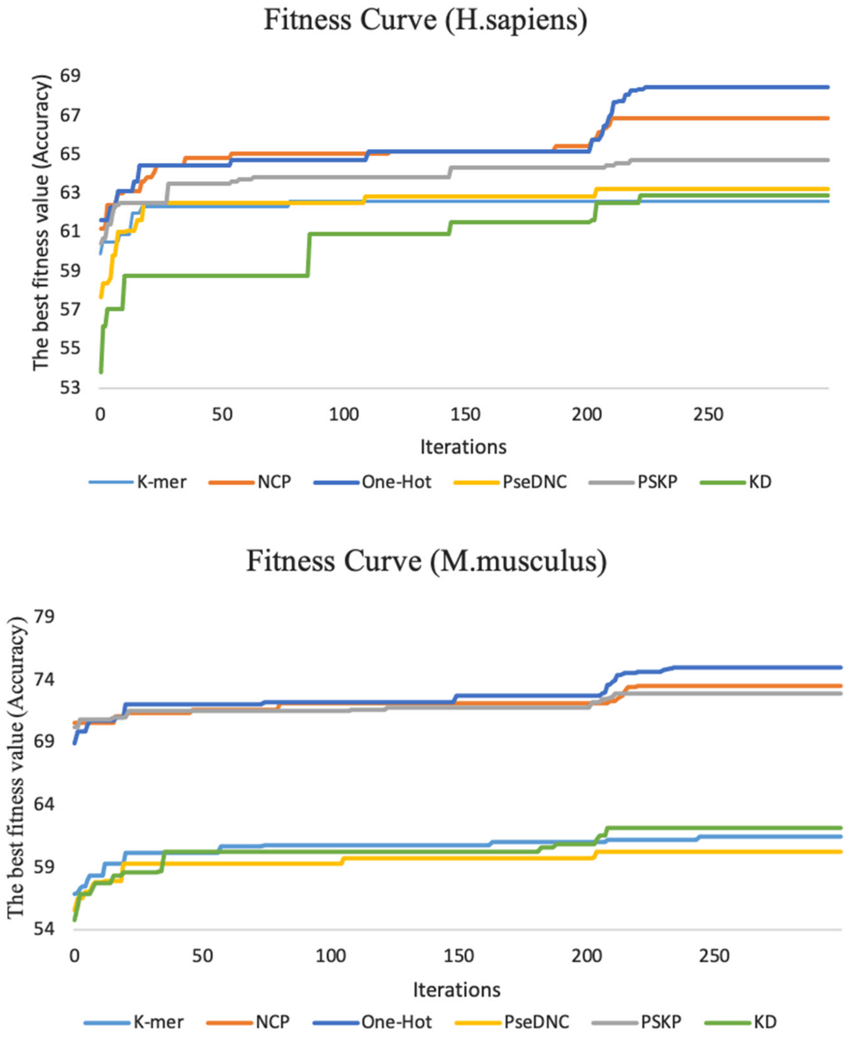

Figure S1. The Fitness curve of six feature representation methods on the H.sapiens and M.musculus datasets

As shown in **Figure S1.**, the feature descriptors of NCP, One-Hot, and PSKP are generally higher than those of K-mer, PseDNC, and KD feature descriptors on the H.sapiens datasets and M.musculus datasets. And the One-Hot feature descriptor has the most obvious and effective enhancement effect. On the H.sapiens datasets, the results are basically the same as those of the S. cerevisiae dataset, and the One-Hot and NCP feature description methods have the strongest representation ability. Compared to the first iteration, the 10-fold cross-validation accuracy of One-Hot and KD feature descriptors improved the most with increasing number of iterations, by 6.9% and 9.0% on the H.sapiens dataset. And the fitness curves on the M.musculus dataset can be found in six feature descriptors basically divided into two levels, One-Hot, NCP and PSKP is a level, and K-mer, PseDNC and KD is a level. The final accuracy of the One-Hot, NCP, and PSKP feature descriptors all exceeded 70%, which was much more than the other three feature descriptors. Overall, it seems that the fitness curves of the six feature descriptors on the three benchmark market datasets can be observed that the accuracy of the 10-fold cross-validation of the basic predictor is improving as the number of iterations increases. It basically indicates that the larger the feature dimension is, the more obvious the improvement effect is, which also shows that the BPSO algorithm plays a very effective role.

Table S1. Comparison between the three feature sets on the four evaluation metrics.

	S. cerevisiae					***H. sapiens***					***M. musculus***				
**Methods**	**ACC**	**MCC**	**SN**	**SP**	**size**	**ACC**	**MCC**	**SN**	**SP**	**size**	**ACC**	**MCC**	**SN**	**SP**	**size**
**K-mer**	0.624	0.25	0.682	0.567	20	0.607	0.22	0.457	0.758	20	0.567	0.13	0.621	0.513	20
**IFS- K-mer**	0.627	0.25	0.701	0.554	14	0.619	0.24	0.636	0.602	4	0.582	0.16	0.597	0.566	6
**PSO- K-mer**	0.653	0.31	0.672	0.634	13	0.626	0.26	0.493	0.760	8	0.614	0.23	0.682	0.547	7
**pseDNC**	0.643	0.29	0.640	0.646	25	0.560	0.20	0.535	0.661	29	0.555	0.11	0.595	0.515	27
**IFS-pseDNC**	0.643	0.29	0.640	0.646	25	0.615	0.25	0.404	0.826	5	0.593	0.19	0.663	0.523	7
**PSO-pseDNC**	0.674	0.35	0.656	0.691	13	0.632	0.27	0.541	0.723	14	0.603	0.21	0.646	0.559	10
**KD**	0.567	0.14	0.637	0.497	61	0.546	0.09	0.560	0.533	41	0.564	0.13	0.593	0.532	41
**IFS-KD**	0.610	0.23	0.764	0.455	10	0.577	0.163	0.721	0.432	16	0.575	0.15	0.636	0.515	3
**PSO-KD**	0.650	0.30	0.697	0.602	26	0.629	0.26	0.624	0.634	17	0.622	0.25	0.676	0.568	18
**PSKP**	0.621	0.25	0.519	0.723	61	0.617	0.24	0.655	0.580	41	0.708	0.42	0.725	0.691	41
**IFS-PSKP**	0.665	0.34	0.576	0.755	14	0.640	0.28	0.657	0.624	21	0.719	0.44	0.754	0.684	18
**PSO-PSKP**	0.672	0.39	0.443	0.901	30	0.647	0.30	0.683	0.612	21	0.729	0.46	0.761	0.697	20
**NCP**	0.654	0.35	0.417	0.892	93	0.629	0.26	0.648	0.610	63	0.693	0.39	0.746	0.640	63
**IFS-NCP**	0.672	0.37	0.481	0.863	12	0.667	0.33	0.687	0.646	32	0.719	0.44	0.786	0.653	9
**PSO-NCP**	0.752	0.52	0.624	0.879	54	0.669	0.34	0.685	0.652	35	0.735	0.47	0.799	0.672	26
**One-Hot**	0.662	0.35	0.487	0.838	604	0.627	0.26	0.665	0.590	404	0.696	0.40	0.775	0.617	404
**IFS-One-Hot**	0.727	0.46	0.682	0.774	139	0.678	0.36	0.642	0.713	50	0.742	0.48	0.782	0.701	85
**PSO-One-Hot**	0.779	0.58	0.650	0.908	314	0.685	0.37	0.677	0.693	192	0.75	0.50	0.807	0.693	192

The **Table S1.** shows the performance of the six feature descriptors on the three benchmark datasets, the four evaluation metrics and the size of each feature descriptor are recorded. From the table, it can be found that the ACC and MCC metrics of the BPSO feature selection method outperform the IFS feature selection method for all three species, as well as both far exceed the

performance of the original features. From the table, it can be found that the ACC and MCC metrics of the BPSO feature selection method outperform the IFS feature selection method for all three benchmark datasets, as well as both far exceed the performance of the original features. In particular, the enhancement effect is most obvious on the S. cerevisiae dataset. In terms of feature size, the BPSO feature selection method selects more balanced size compared to the IFS features selection method. The IFS method selected relatively low-dimensional features as the optimal features on the K-mer, PseDNC feature descriptions of the H. sapiens dataset and on the K-mer, KD, and NCP feature descriptors of the M. musculus dataset. It was also found in the later experiments that the generalization ability of the basic predictors trained on these feature descriptions were lower than that of the basic predictors trained by the BPSO feature selection method.

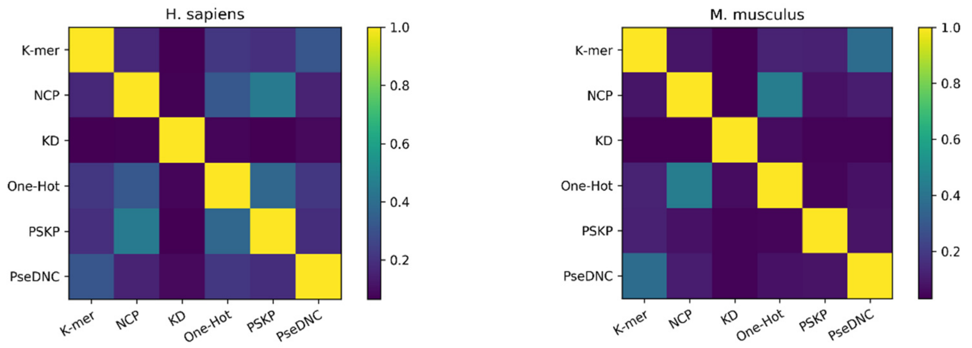

Figure S2. Pearson Correlation Coefficients of the feature descriptors on the H.sapiens and M.musculus species

Figure S2. shows the heatmap of Pearson correlation coefficients for the six feature descriptors on the H.sapiens dataset and the M.musculus dataset. Where the shade of color indicates the correlation between the basic predictors, the lighter the color, the stronger the correlation and the less differences in the predictors. It can be found that the correlation between the KD feature descriptor and the other five feature descriptors is less than 0.2, both in the H.sapiens dataset and in the M.musculus dataset. The correlation between PSKP and the One-Hot, NCP descriptors is also very low at less than 0.3 in the M.musculus dataset. And all correlations between individual features are basically lower than 0.5, so it can basically indicate that there is a strong variability between these features. For more detailed results, refer to Figure S3.

Table S2. Comparison of the 10-fold cross-validation scores of PsoEL-PseU with existing state-of-the-art pseudouridine site predictors.

Species	Predictor	ACC	MCC	SN	SP
S. cerevisiae	IRna-PseU	0.645	0.29	0.647	0.643
	PseUI	0.641	0.29	0.647	0.643
	iPseU-CNN	0.682	0.37	0.664	0.705
	XG-PseU	0.682	0.37	0.668	0.695
	RF-PseU	0.748	0.49	0.772	0.724
	PsoEL-PseU	0.803	0.62	0.691	0.914

H. sapiens	IRna-PseU	0.604	0.21	0.61	0.598
	PseUI	0.642	0.28	0.649	0.636
	iPseU-CNN	0.66	0.34	0.65	0.68
	XG-PseU	0.661	0.32	0.635	0.687
	RF-PseU	0.643	0.29	0.661	0.626
	PsoEL-PseU	0.708	0.42	0.669	0.747
M. musculus	IRna-PseU	0.691	0.38	0.733	0.648
	PseUI	0.704	0.41	0.799	0.703
	iPseU-CNN	0.718	0.44	0.748	0.691
	XG-PseU	0.72	0.45	0.765	0.676
	RF-PseU	0.748	0.50	0.731	0.765
	PsoEL-PseU	0.765	0.53	0.822	0.708

Table S2 demonstrates the comparison of the 10-fold cross-validation score of PsoEL-PseU with the current state-of-the-art pseudouridine site predictor. The results show that our proposed predictor PsoEL-PseU outperforms all the current top predictors by a wide margin. on the S. cerevisiae dataset, the accuracy exceeds 5.5% compared to the RF-PseU predictor, surpassing more than 80% for the first time. On the H. sapiens dataset, PsoEL-PseU also outperforms the XG-PseU, which has the highest accuracy, by 4.7%, also exceeding 70% for the first time. And on the M. musculus dataset, the accuracy values range from 1.7% to 7.4% improvement compared to the other state-of-the-art pseudouridine site predictors. From the ACC and MCC in the Table S2, basically indicate that the performance of our proposed predictor PsoEL-PseU has reached the top level at present.
